# An efficient and accurate solver for large, sparse neural networks

**DOI:** 10.1186/1471-2202-16-S1-P179

**Published:** 2015-12-18

**Authors:** Roman M Stolyarov, Andrea K Barreiro, Scott Norris

**Affiliations:** 1 Department of Mathematics, Southern Methodist University, Dallas, TX, USA; 2 Harvard-MIT Department of Health Sciences and Technology, Cambridge, MA, USA

## 

The mammalian brain has about 10^11 ^neurons and 10^14 ^synapses, with each neuron presenting complex intra-cellular dynamics. The huge number of structures and interactions underlying nervous system function thus make modeling its behavior an extraordinary computational challenge. One strategy to reduce computation time in networks is to replace computationally expensive, stiff models for individual cells (such as the Hodgkin-Huxley equations and other conductance-based models) with integrate-and-fire models. Such models save time by *not *numerically resolving neural behavior during its action potential; instead, they simply detect the occurrence of an action potential, and propagate its effects to postsynaptic targets appropriately. Thus, a complicated system of continuous ordinary differential equations is replaced with a simpler, but *discontinuous*, differential equation.

However, accurate existing methods for integrating discontinuous ordinary differential equations (ODEs) scale poorly with problem size, requiring O(N^2^) time steps for a system with N variables. The underlying challenge is that discontinuities introduce O(dt) errors to conventional time integration schemes, thus requiring very small time steps in the vicinity of a discontinuity [1].

**Figure 1:**
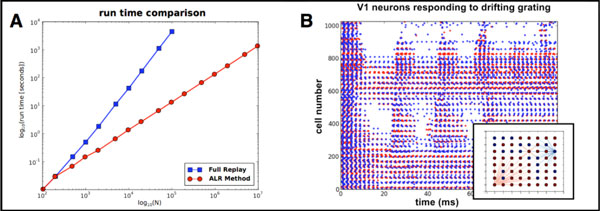
**(A) Comparison of runtime for a fully event-driven ("Full Replay") and ALR methods, for integrate-and-fire networks of various system sizes N**. (B) Raster plot of a 32 × 32 grid of V1 model neurons responding to a drifting grating stimulus. Inset: schematic of a subset of the network, with selected synapses identified and shaded by strength. Red: AMPA; orange: NMDA, blue: fast GABA.

In this work, we propose a method to reduce this computational load by embedding local network "repairs" within a global time-stepping scheme. In addition, high-order accuracy can be achieved without requiring the global time step to be bounded above by the minimum communication delay, as is currently required in the hybrid time-driven/event-driven scheme used by NEST [2]: this allows more powerful exploitation of exact subthreshold [3,4] and quadrature-based [5] integration schemes. If the underlying network is sufficiently sparse the algorithm, *Adaptive Localized Replay *(ALR), will attain time complexity O(N) (Figure 1A). We apply our method to a network of integrate-and-fire neurons that simulates dynamics of a small patch of primary visual cortex (Figure 1B) [5,6].
